# The comparative effect of propolis and chlorhexidine mouthwash on oral nitrite-producing bacteria and blood pressure regulation

**DOI:** 10.1080/20002297.2024.2439636

**Published:** 2024-12-16

**Authors:** R. Bescos, L. du Toit, A. Redondo-Rio, P. J. Warburton, T. L. Nicholas, M. Kiernan, R. A. Burton, L. Belfield, G. Montagut, A. Benavente, W. Vevers, T. Gabaldón, Z. Brookes, P. Casas-Agustench

**Affiliations:** a School of Health Professions, Faculty of Health, University of Plymouth, Plymouth, UK; b Barcelona Supercomputing Centre (BSC-CNS), Barcelona, Spain; c Institute for Research in Biomedicine (IRB Barcelona), Barcelona, Spain; d School of Biomedical Sciences, Faculty of Health, University of Plymouth, Plymouth, UK; e Peninsula Dental School, Faculty of Health, University of Plymouth, Plymouth, UK; f Brunel Medical School, College of Health and Life Sciences, Brunel University, England, UK; g School of Biological and Marine Sciences, Faculty of Science and Engineering, University of Plymouth, Plymouth, UK; h Catalan Institution for Research and Advanced Studies (ICREA), Barcelona, Spain; i CIBER de Enfermedades Infecciosas, Instituto de Salud Carlos III, Granada, Spain

**Keywords:** Propolis, chlorhexidine, microbiome, nitrate, nitrite, nitric oxide, blood pressure

## Abstract

**Background:**

Propolis mouthwash (PROP-M) has demonstrated antibacterial properties like those of chlorhexidine mouthwash (CHX-M). However, its impact on the abundance of oral nitrite-producing species (NPS) and nitrite-producing activity (NPA) remains unexplored.

**Methods:**

Forty-five healthy individuals were randomised into 2 groups to rinse their mouth twice a day for seven days with either CHX-M (*n* = 21) or PROP-M (*n* = 24). Metagenomic sequencing (16S rRNA) was performed on saliva samples collected before and after each treatment. Additionally, salivary biomarkers and blood pressure were measured.

**Results:**

CHX-M increased the relative abundance of NPS (*p* < 0.001) but significantly impaired the NPA (*p* < 0.001) compared to baseline and PROP-M. No significant differences in the relative abundance of NPS and NPA were observed in the PROP-M group. However, a significant increase of plasma nitrate (+7 µmol/L, *p* = 0.047) and a decrease in systolic BP (−2 mmHg, *p* = 0.022) was observed in this group compared to the baseline.

**Conclusion:**

The results indicate that PROP-M had a smaller effect on the abundance of NPS and NPA compared to CHX-M. Additionally, PROP-M reduced blood pressure in healthy individuals, but this effect was not associated with changes in the oral microbiome.

## Introduction

Chlorhexidine (CHX) is an antimicrobial agent that has been used in dentistry for over 40 years due to its clinical effectiveness against pathogenic bacteria [1]. Current dental guidelines recommend short-term (2–4 weeks) adjunctive use of CHX in specific cases, alongside oral hygiene measures, to help patients manage gingivitis and periodontitis [2]. However, recent evidence [3–7] suggests that CHX mouthwash (CHX-M) (0.2%) can inhibit nitrite-producing species (NPS) [3]. Although the majority of salivary nitrite likely originates from oral nitrate , a more accurate term for species producing nitrite would be NPS, as other pathways such as the oxidation of nitric oxide (NO) and ammonium can also contribute to nitrite production [8]. Oral NPS include representatives of *Neisseria, Rothia, Veillonella, Actinomyces, Corynebacterium, Haemophilus and Kingella*, constituting nearly 20% of all measured genera in the oral cavity [9,10]. They are important for maintaining a healthy salivary pH by producing nitrite and ammonium and metabolising lactic acid as a carbon source during denitrification [3,10].

Following the bacterial conversion of nitrate into nitrite, nitrite is swallowed and further converted into NO in the acidic environment of the stomach, a reaction associated with blood pressure reduction [11,12]. Some nitrite can also enter the bloodstream, where it can be further reduced to NO through several pathways, contributing to additional blood pressure regulation [13]. Inhibition of NPA by CHX mouthwash (CHX-M) has been shown to reduce salivary nitrite availability, which has been associated with elevated blood pressure by some studies [3–5,14], but not all [15,16]. Concerns have also emerged about the use of CHX-M, particularly its association with increased mortality risk in critically ill patients [17–19]. Moreover, long-term use of CHX has been linked to minor side effects such as altered taste, teeth and tongue staining and burning sensation [1]. Thus, there is a growing need for alternative mouthwashes based on natural products that can manage oral health while preserving beneficial nitrite-producing bacteria, which play an essential role in supporting both oral and cardiovascular health [20].

Propolis (PROP) is a non-toxic resinous substance produced by honey bees, which is rich in flavonoids and phenolic compounds with antibacterial properties [21]. In recent years, its use as a mouthwash (PROP-M) has gained attention, showing similar efficacy to CHX-M in reducing dental plaque and inhibiting potential oral pathogens [22–25]. Moreover, PROP supplementation has shown promising effects in reducing BP in rats [26,27] and hypertensive individuals [28]. This effect could be attributed to increase NO availability due to the upregulation of endothelial NO synthase (eNOS) expression [29]. Studies in rodents [30,31] have also reported positive changes in the gut microbiome, including higher abundance of *Lactobacillus* species and a lower *Bacteroidetes*/*Firmicutes* ratio following an enriched diet with PROP. However, no study has investigated the impact of PROP-M on the oral microbiome, NPS and BP control.

This study aimed to compare the effects of CHX-M and PROP-M on NPA and abundance of NPS, salivary and plasma biomarkers and BP in healthy individuals. We hypothesised that PROP-M would preserve or elevate the NPA and abundance of NPS, and this would be accompanied by an increase of salivary and plasma nitrite and a lower BP. In contrast, CHX-M would significantly reduce the NPA and abundance of NPS, and this will be associated with lower salivary and plasma nitrite levels and elevated BP.

## Methods

### Study design and sample size

This study was simple randomised and double-blind (provider and participant). The study population consisted of healthy adults (18–50 y/o) with a BMI <30 kg/m^2^ and no following periodontal treatments or using antimicrobial products and antibiotics within 3 months before initiation of study. All participants provided informed consent prior to the initiation of the study. The study was approved by the Human Ethics Committee of Plymouth University (16/17–666) and registered in ClinicalTrials.gov (NCT04117451).

Based on previous data with CHX-M and using an online sample calculator for two independent groups (ClinCalc.com), we estimated that 50 individuals (25 in each group) were required to detect a 20% difference in NPA (200 µM nitrite at baseline vs. <160 or >240 µM post-treatment) with 80% statistical power and a standard α-value of 0.05 [3].

### Propolis (PROP) and chlorhexidine (CHX) mouthwash

Raw bee PROP was obtained from the Prades mountains (Tarragona, Spain). Pure bioactive compounds of PROP were extracted by mixing it with 70% ethanol for 72 hours in a 1:10 (weight/volume) ratio. Then, PROP was filtered using a microfiltration apparatus (filter size 50 mm). A second extraction using the same volume (ethanol/water) and time (72 h) was performed with the remaining PROP from the first extraction before to filter it. The extract of PROP obtained from both extractions was mixed in a 2 L sterile glass flask. Then, the ethanol was evaporated using a Rotary Evaporator (Buchi Rotavapor *R*-3, Switzerland). Once all the ethanol was removed, ultrapure water was added to obtain a final concentration of 2.5% (0.4 mg in 10 mL) of PROP extract.

The phenolic composition of the final propolis solution was analysed using ultra high-performance liquid chromatography (DIONEX UltiMate 3000 UHPLC, Thermo Scientific Inc.), equipped with a UV detector set at 277 nm (DIONEX MWD-3000). The volume of injection was 10 μL per sample at 25°C. The chromatographic separation was obtained using a C-18 reverse-phase column at 25°C (BDS Hypersil 25 cm × 4.6 mm, 5 μm, protected by a C18 Uniguard cartridge, Thermo Scientific). The mobile phase consisted of two components: acetonitrile with 0.1% formic acid (solvent A); and 0.1% formic acid in Milli-Q water (solvent B) with a flow rate of 1 mL/min using the following gradient: 10% A for 5 min increasing to 100% A over 25 min, decreasing from 100% to 10% between 35 min and 40 min and remaining at 10% for the next min. All standard phenolic compounds (dihydroxy benzoic acid, syringic acid, p-coumaric acid main, benzoic acid, trans-cinnamic acid) were purchased from Sigma and diluted in 10% acetonitrile in Milli-Q water (Fisher Scientific). For additional details about this method refers to the supplementary material.

CHX-M was prepared using a commercial product (0.2% CHX, Corsodyl Mint, GlaxoSmithKline, UK). The pharmaceutical company had no participation in this study, nor did it provide any funding or material support.

Sterile falcon tubes were filled with 10 mL of this PROP mouthwash (PROP-M) and chlorhexidine mouthwash (CHX-M) (Corsodyl, 0.2%, GlaxoSmithKline, UK) and covered with aluminium foil to avoid degradation and identification of treatment and stored at −20°C.

### General protocol

All participants completed a medical questionnaire prior the start of the study to provide information about their periodontal history. Additionally, a subgroup of participants (CHX-M = 13; PROP-M = 12) underwent an oral examination before and after the treatment by a qualified dentist. Due to COVID-19 restrictions, the oral examination could not be conducted on the remaining participants, so we relied on medical questionnaires to assess their periodontal health. The periodontal examination included an oral soft tissue examination (noting any anomalies), recording of decayed, missing teeth (DMFT), recording of sextant basic periodontal examination (BPE) scores using a BPE probe. Only participants with a BPE of 2 or lower, but no more than one BPE 2 in any sextant were included in this study.

Participants meeting the inclusion criteria attended the laboratory on two different occasions under fasting conditions (>3 h). They were randomly assigned by a researcher using a random-numbers table (with blocks of 10 and an allocation ratio of 1:1) into one treatment: 1) CHX-M or 2) PROP-M. Participants rinsed (10 mL) their mouth twice a day (morning and evening) for one week. The duration of the protocol was based on previous research showing positive effects of PROP-M in the clinical management of gingivitis [24]. A non-stimulated saliva sample (3 mL) was collected into a Falcon sterile tube and rapidly centrifuged at 16,200 × g and 4°C for 10 min. The supernatant was transferred to a sterile Eppendorf tube and stored at −80°C for biochemistry analyses. The pellet at the bottom of the tube was also stored at −80°C for metagenomic analyses. The NPA was measured by holding a mouth rinse (10 mL) with sodium nitrate (80 µmol) for 5 min. Then, the mouth rinse was collected into a Falcon sterile tube and centrifuged at 2,558 × g and 4°C for 10 min. The supernatant was collected and stored at −80°C prior to measure the nitrite concentration. NPA was calculated as follows: NPA = Total nitrite in the mouth rinse – salivary nitrite under resting conditions. A blood sample was collected from an antecubital vein using lithium-heparin tubes (BD Vacutainer®, Becton Dickinson, Plymouth, UK) and rapidly centrifuged at 2,878 × g and 4°C for 10 min. Then, the plasma was transferred to a sterile Eppendorf tube and stored at −80°C for biochemistry analyses.

Following the collection of samples, body height, weight and body fat were measured using a stadiometer (Seca, Birmingham, UK) and bioimpedance analyser (Tanita, TBF-300 MA, Tokyo, Japan), respectively. BP was measured in triplicate using an electronic BP monitor (Connex ProBP 3400, Welch Allyn UK) after resting for 10 min. The second and third readings were averaged to determine mean BP. Finally, a flow-mediated dilation test was performed on the left arm to analyse the microvascular function as described previously [14]. Briefly, levels of oxygenated haemoglobin (HbO_2_) and deoxyhaemoglobin (HHb) on the left forearm (extensor digitorum) were continuously recorded using a NIRS device (NIRO-200NX, Hamamatsu, Japan) at an output frequency of 1 hz. After baseline measurements (2 min), an automatic pneumatic cuff (Hokanson E-20 AG101, USA) was inflated ~5 cm above the elbow for 5 min to an occlusion pressure of 200 mmHg. Then, inflation of the cuff was rapidly released (<1 second) and the NIRS measurements were continuously monitored for 5 more min. Several NIRS measurements were analysed (supplementary material). Participants returned to the laboratory a week after to undertake the same measurements in the same order.

## Laboratory analyses

### Blood and saliva analyses

Salivary and plasma nitrate and nitrite were analysed using high-performance liquid chromatography (HPLC) device (ENO-30; EiCom, Kyoto, Japan) [14]. Briefly, 100 µL of saliva and plasma were diluted at 1:10 and 1:1, respectively, with carrier solution (containing 10% methanol, 0.15 M NaCl/NH4Cl, and 0.5 g/L 4Na-EDTA) and methanol before injecting 10 μL into the HPLC system. A standard curve was generated by injecting 10 μL of nitrate and nitrite solutions (0.1 μM, 31.2 μM, 250 μM and 500 μM).

Glucose and lactate levels were analysed using a biochemistry analyser (YSI 2500 Stat Plus, YSI Life Sciences, USA). The pH was measured using digital pH meter (Lutron Electronic Enterprise Co Ltd., Model PH-208, Taiwan) that was calibrated according to the manufacturer’s instructions. The buffering capacity was analysed by mixing 250 µL of saliva and plasma with 750 µL of HCl (3 mmol/L) and shaken for 20 min. The pH was then measured using the same pH meter as above.

Ammonia levels in saliva were measured using an assay kit (Merck Life Science AA0100, Gillingham, UK). Briefly, 100 µL of saliva were diluted at 1:10 with ultrapure water. Then, 1 mL of ammonia standard solution was added and incubated for 5 min at room temperature. Absorbance was read at 340 nm using a spectrometer (Camspec M508, Leeds, UK). Following this, 10 µL of L-glutamate dehydrogenase was added to the sample, and it was incubated for an additional 5 min before measuring the final absorbance at 340 nm.

### Oral microbiota

#### DNA extraction and sequencing

Saliva pellets were used for the analysis of the microbiome. DNA extraction was performed with the ZymoBIOMICS 96 MagBead DNA kit (ref #D4302, ZymoResearch), using the FastPrep-96 and 32-PurePrep (MolGen). Sequencing was performed in the Illumina MiSeq using v3 chemistry. For additional details about the extraction and sequencing of the oral microbiome, refer to the supplementary material.

#### Antimicrobial activity

The antibacterial activity of PROP-M and CHX-M against *Rothia dentocariosa* (DSM 43,762) and *Streptococcus mutans* (NCIMB 702,062) were also analysed in liquid bacterial cultures.

## Statistical analyses

Results are presented as mean ± standard error of the mean (SEM), except when the standard deviation (SD) was used, as indicated in the results. The Shapiro–Wilk test was used to assess data distribution. Differences between both groups at baseline were analysed using a non-paired t-test or a Wilcoxon rank-sum test according to the distribution of the data. A two-way repeated measures ANOVA was performed to assess the main effects and interaction between treatments (CHX and PROP mouthwash) and time (pre and post treatment). When the ANOVA revealed a significant interaction, specific differences were identified using individual comparisons. Analysis was carried out using the SPSS software (SPSS Statistics, IBM® Version 24) and statistical significance was taken as *p* < 0.05.

Microbial community analyses were performed with R v4.3.1 and Rstudio v2023.06.0.421. R package dada2 v1.12.1 [32] was used for sequence quality filtering, with parameters ‘truncLen = c(270, 225), trimLeft = 10 minLen = 50, maxEE = 8, maxN = 0’. Dada2 was also used for ASV clustering, chimera removal and taxonomic assignment of ASVs with database SILVA nr99 v138.1 [33]. Phyloseq v1.44.0 [34] was then used to analyse the abundance tables. To reduce noise, ASVs present in less than 20 samples were removed, considering only samples with more than 50 reads of that given ASV. Because of the compositional nature of metagenomic data [35], abundance tables were 0-replaced using the CZM method with R package zCompositions v1.4.0.1 [36] and then CLR-transformed with package CoDaSeq v0.99.6. Principal Component Analysis (PCA) was performed with the *prcomp* function in package stats v4.3.1, and correlation of metadata with the principal components was tested with a Welch’s F-test. Alpha diversity measures were compared using Wilcoxon and Wilcoxon signed-rank tests. Beta diversity analyses were based on a PERMANOVA test on Aitchison’s distance, calculated with package robCompositions v.2.3.1 [37]. Differential abundance analyses were performed using mixed-effect linear models with R packages lmer4 v1.1.33 [38]. Sequencing batch, sex and age were passed on to the linear models as fixed effects in all cases. When comparing paired samples, the subject was added as a random effect. P-values for the multiple tests were adjusted using the Benjamini–Hochberg correction (FDR correction), and only correlations with an adjusted p-value <0.05 were considered significant and reported. For the analysis of NPS, read counts of NPS and genera were identified based on the classification by Rosier *et al* [10]. (supplementary material). The summed read counts were treated as a single taxon for downstream analyses. Additionally, we analysed changes in the relative abundance of bacterial species associated with periodontitis [39] (supplementary material).

## Results

Forty-eight participants participated in the study between June 2019 and December 2022. Three participants (CHX-group) dropped out due to illness. Two subjects had to be excluded from the microbiome analyses (1 CHX-M and 1 PROP-M) due to the lack of the saliva pellet. The main characteristics of the participants are shown in Table 1. Three participants in the CHX-M group reported a feeling of burning mouth after the treatment, but no major adverse events were reported by any participant.

**Table 1.** Main characteristics of the participants in the chlorhexidine (CHX-M) and propolis (PROP-M) group.

	CHX-M (n = 21)	PRO-M (n = 24)
Subjects (F:M)	21 (17:4)	24 (19:5)
Age (y)	26 ± 1.4	25 ± 1.4
Weight (kg)	61.6 ± 2.4	63.5 ± 2.9
Height (m)	1.68 ± 0.02	1.68 ± 0.02
Body mass index (kg/m^2^)	21.5 ± 0.6	22.4 ± 0.6
Body fat (%)	21.6 ± 1.5	23.8 ± 1.4

### Nitrite-producing activity (NPA) and abundance of nitrite-producing species (NPS)

**Figure 1.**
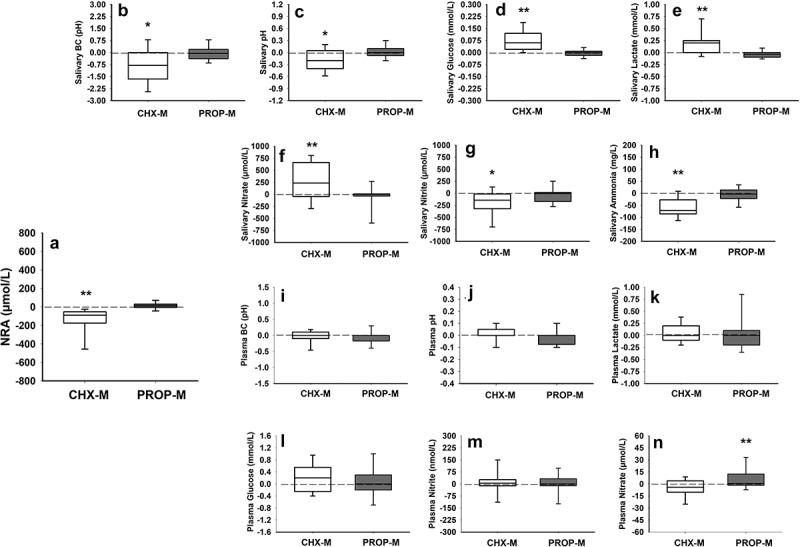
Changes (delta) (mean ± SD) in nitrite-producing activity (NPA) of oral bacteria (2A) and saliva buffering capacity (BC) (2B), pH (2C), glucose (2D), lactate (2E), nitrate (2F), nitrite (2 G), ammonia (2 h), and plasma buffering capacity (BC) (2I), pH (2J), lactate (2K), glucose (2 L), nitrite (2 M), nitrate (2N) after the chlorhexidine (CHX-M, *n* = 21) and propolis (PROP-M, *n* = 24) treatment.

**Figure 2.**
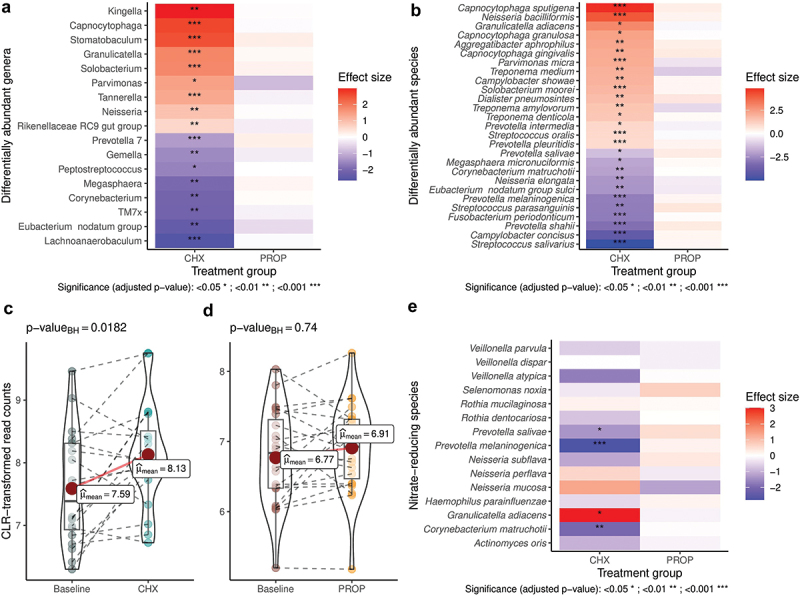
Differences in bacterial abundance of classified genera and species after the chlorhexidine (CHX-M, *n* = 20) and propolis (PROP-M, *n* = 23) treatment (2A). Differences in nitrite-producing species between baseline levels and after the CHX-M (2B) and the PROP-M (2C) treatment. Differential abundance of individual nitrite-producing species after both treatments using linear models (2D).

At baseline, NPA was similar in both groups (*p* > 0.05). After treatment, a significant reduction was observed in the CHX-M group compared to baseline (*p* < 0.001) and the PROP-M group (*p* < 0.001) (Figure 1). Out of the 52 NPS, 25 were identified in our dataset. A significant increase in the relative abundance of NPS was observed in the CHX-M group compared to baseline and the PROP-M group (*p* < 0.001) (Figure 2). The high-throughput sequencing data will be available within the NCBI Sequence Read Archive (SRA) database: PRJNA1088292.

(*represents statistical differences between pre- and post-measurements within the same group; **represents statistical differences between both groups after treatment).

### α-diversity and β-diversity

**Figure 3.**
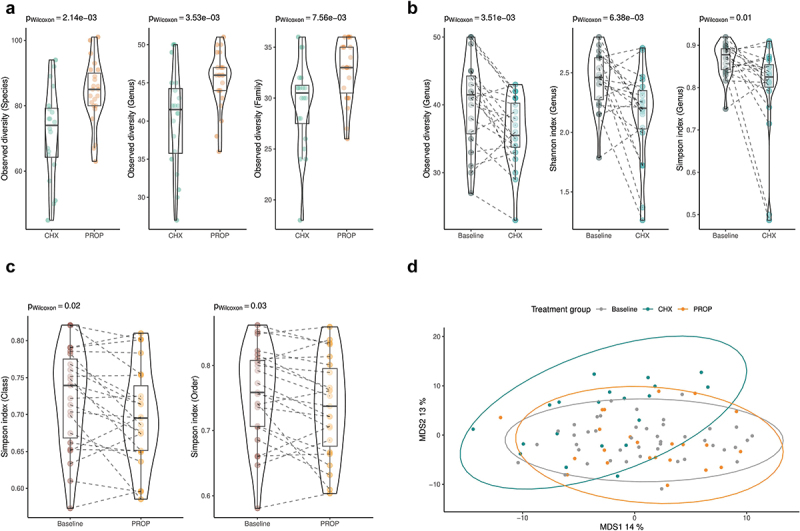
Observed diversity differences at species, genus and family level in the chlorhexidine (CHX-M, *n* = 20) and propolis (PROP-M, *n* = 23) group at baseline (3A). α-diversity (Shannon and Simpson index) in the CHX-M group before and after the treatment (3B). α-diversity (Simpson index) at class level in the PROP-M group before and after the treatment (3C). MDS representation of β-diversity (Aitchinson’s distance) at genus level in the CHX-M (green), PROP-M (orange) and at baseline (gray).

At baseline, the PROP-M group showed higher α-diversity compared to the CHX-M group (Figure 3, *p* < 0.01). After treatment, a major decrease in α-diversity (Shannon and Simpson indexes, *p* < 0.005) was observed in the CHX-M group, while α-diversity was less altered in the PROP-M with some decreases in the Simpson index at high taxonomic levels (*p* < 0.05). After treatment, a significant change in β-diversity was observed in the CHX-M group (*p* < 0.01) compared to baseline.

### Differential bacterial abundance

At baseline, the genus *Neisseria* was more abundant in the CHX-M group. After the treatment, changes in the abundance of 17 genera (42 species) were observed in the CHX-M group, while no significant differences were observed in the PROP-M group (Figure 2). Additionally, a significant increase was observed in the *Firmicutes*/*Bacteroidetes* ratio in the CHX-M group (*p* = 0.001), while a significant decrease was observed in the PROP-M group (*p* = 0.003).

**Figure 4.**
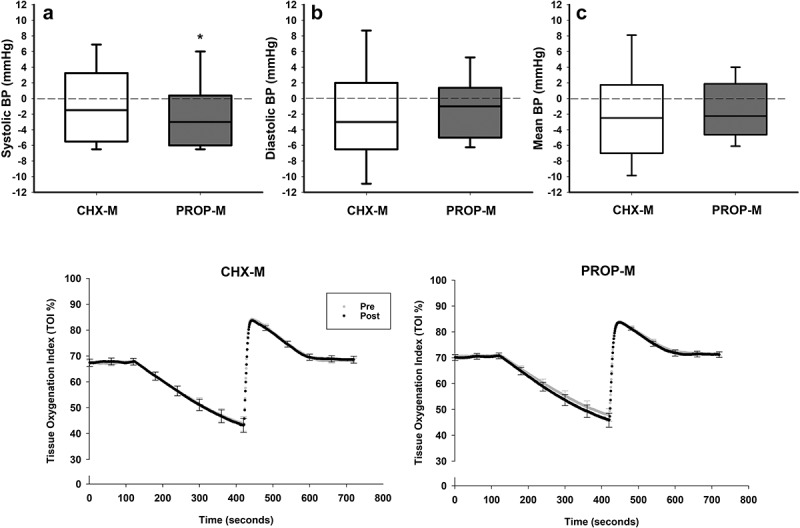
Top: changes (delta) in systolic (SBP) (4A), diastolic (DBP) (4B) and mean arterial (MAP) (4C) blood pressure after the chlorhexidine (CHX-M, *n* = 21) and propolis (PROP-M, *n* = 24) treatment.

Regarding the relative abundance of bacterial species related to periodontal disease, a significant increase was observed in these species in the CHX-M group after the treatment (*p* = 0.001), but this was not observed in the PROP-M group (Figure 4).

Bottom: Microvascular response (mean ± SEM) to a hyperaemia reactive test at baseline (Pre: grey line) and after (Post: black line) the chlorhexidine (CHX-M, *n* = 21) and propolis (PRO-M, *n* = 24) treatment.

(*represents statistical differences (*p* < 0.05) between pre and post measurements within the same treatment).

### Salivary biomarkers

At baseline, all the salivary biomarkers were similar between both groups (*p* > 0.05). After treatment, a reduction in pH (*p* = 0.005), nitrite (*p* = 0.006), buffering capacity (*p* = 0.004) and ammonia levels (*p* < 0.001) was observed in the CHX-M group (Figure 1). Furthermore, an increase in nitrate (*p* < 0.018), lactate (*p* = 0.029) and glucose (*p* < 0.001) was observed in the CHX-M group compared to baseline. No changes in salivary markers were observed in the PROP-M group after the intervention.

Comparison between both groups indicated a higher salivary nitrate (*p* < 0.007), lactate (*p* = 0.005) and glucose (*p* < 0.001) concentration, as well as lower ammonia levels (*p* < 0.023) in the CHX-M group after the treatment (Figure 1).

### Plasma biomarkers

At baseline, nitrite, nitrate, pH, buffering capacity, lactate and glucose were similar in both groups (*p* > 0.05). After treatment, a significant increase in plasma nitrate was observed in the PROP-M group (*p* = 0.047) (Figure 1).

### Blood pressure and microvascular function

At baseline, systolic and mean BP were similar (*p* > 0.05) between both groups, however, diastolic BP was significantly lower in the PROP-M group (61.3 ± 0.9 mmHg), compared to the CHX-M group (65.7 ± 1.6 mmHg; *p* = 0.018). After treatment, systolic BP in the PROP-M group was lower compared to baseline (*p* = 0.022), but there were no differences (*p* > 0.05) in this parameter between both groups (Figure 4).

At baseline, all microvascular parameters were similar between both groups. After treatment, a lower ∆min/baseline ratio (the difference between baseline and the minimal value reached during the ischemia phase) was observed in the PROP-M group, but this was not statistically significant (*p* = 0.078) (Figure 4).

### Periodontal health

At baseline, plaque and bleeding scores were similar in both groups (*p* > 0.05). After treatment, a significant reduction of plaque (*p* = 0.005) and bleeding (*p* = 0.023) scores occurred in the CHX-M group compared to baseline. Changes in plaque (*p* = 0.131) and bleeding (*p* = 0.155) scores were not observed in the PROP-M group. For additional details refer to the supplementary material.

### Phenolic compounds and antibacterial activity (in vitro)

Six polyphenols were detected in the PROP-M including shikimic acid, quinic acid, gallic acid, benzoic acid, quercetin, kaempferol and phloroglucinol. For additional details refer to the supplementary material. The growth of *S. mutans* and *R. dentocariosa* was inhibited with CHX-M (0.2%) and PROP-M (20%) over a 24-hour period.

## Discussion

This is the first study investigating the effects of a PROP-M on oral NPS and NPA. Our findings indicate that neither the composition of the oral microbiome nor the NPA were significantly altered by the PROP-M, which is consistent with our original hypothesis anticipating the preservation or potential increase in NPS abundance or NPA following PROP-M use. This hypothesis was based on previous studies showing positive changes in the composition of the gut microbiome when PROP was orally administered to rodents [30,31]. The absence of major microbial changes in our study may raise the question of whether PROP-M had antibacterial properties. To address this, we conducted an *in-vitro* experiment to assess the inhibitory effect of PROP-M against *S. mutans* and *R. dentocariosa*. PROP-M effectively inhibited the growth of both bacterial strains at a concentration of 20%, though not at lower concentrations. Importantly, this concentration was eight times higher than that used in our PROP-M formulation (2.5%). We selected this concentration based on commercially available PROP-M formulations and previous studies that used similar concentrations [40]. Additionally, the consumption of propolis at comparable concentrations (2%) has been reported to modify the gut microbiome in mice [30]. This concentration was over twelve times higher than the CHX-M concentration used in this study (0.2%). However, our findings, along with recent evidence [41], suggest that higher propolis concentrations may be required for PROP-M to achieve significant modulatory effects of the microbiome. This could be important when considering PROP-M as a potential alternative to CHX-M in terms of antimicrobial efficacy.

The results from our current study regarding CHX-M are consistent with previous findings, which demonstrated a significant reduction in NPA and a decrease in the oral microbiome diversity [3]. These changes were accompanied by lower abundance of *Prevotella* and a higher abundance of *Neisseria* and *Streptococcus* species [3]. However, contrary to our hypothesis, we observed a significant increase in the relative abundance of NPS in the CHX-M group. A possible explanation for this discrepancy is that CHX-M may have reduced the overall bacterial load as indicated in previous studies [42]. It is important to note that our metagenomic analyses (16S rRNA) measured the relative bacterial abundance within the microbiome composition, not the total bacterial count in saliva. This highlights a key limitation of studies focusing exclusively on microbiome composition, which may not capture functional shifts accurately. For example, a relative abundance of 30% from a bacterial population of 10^7 (3 million) is still lower than 15% of a population of 10^8 (15 million). Although we did not measure total bacterial load in this study, this is a crucial factor to consider in future studies. Additionally, the lower NPA observed in this study despite the increase in the relative abundance of NPS in the CHX-M group could be due to inhibition of the nitrate-reduction enzymes. This is another important question that remains to be addressed in future studies.

The reduction in NPA of oral bacteria after using CHX-M can significantly alter salivary composition and pH regulation. Like in previous studies [3,14], we found a decrease in salivary nitrite and ammonia levels, alongside an increase in lactate and glucose concentrations after using CHX-M. CHX-M has been shown to disrupt glucose metabolism in species like *Streptococcus*, leading to incomplete glucose utilization [43]. Furthermore, CHX-M can reduce the total bacterial load [42], resulting in fewer bacteria capable of metabolising lactate, thereby leaving higher residual levels in saliva. Supporting this, CHX-M reduced the relative abundance of some lactate-consuming bacteria such as *Veillonella parvula*, *Veillonella atypica* and *Megasphera* [10]. Thus, changes in salivary composition in the CHX-M group may reflect a reduction in microbial activity and shift in the types of bacteria present in the oral cavity.

Elevated salivary lactate and glucose levels in the CHX-M group were associated with a decrease in salivary pH and buffering capacity. This is relevant, as lower salivary pH and buffering capacity are strongly linked to an increased risk of dental caries [44]. In terms of oral health, our results also revealed that CHX-M, but no PROP-M, significantly increased the relative abundance species associated with periodontitis [39]. Although the dental use of CHX-M has traditionally been justified by its effectiveness in reducing dental plaque and bleeding, an effect we also observed in this study, more caution is needed regarding its potential to disrupt the microbial balance in the oral cavity. It must be noted that we used 0.2% CHX-M, which is available over the counter in the UK and Europe and is recommended for short-term intensive plaque control [45]. In contrast, in the United States, CHX-M is prescribed at 0.12%, while concentrations of 0.06% are referred to as daily rinses [45]. However, the effect of CHX-M concentrations below 0.2% on the oral microbiome remains largely unknown.

Furthermore, lower NPA of oral bacteria can also challenge BP control by reducing lower NO availability. In support of this, several studies, but not all [4,15,16], have reported an increase in BP after using CHX-M in healthy [3,5,14] and hypertensive individuals [4]. In the current study, we did not observe a BP increase in the CHX-M group, which aligns with the findings by Sundqvist et al [16] in a group of young healthy females. Perhaps, in this type of population, vascular eNOS may be rapidly modulated to increase its activity in response to lower nitrite availability [16]. This compensatory mechanism could preserve vascular NO generation and nitrite levels, thereby preventing a BP increase. While this explanation seems plausible, further research in different populations is needed to better understand the role of NPS in BP control.

In the PROP-M group, we observed a small but significant reduction in systolic BP. Additionally, forearm oxygenation levels (desaturation slope) appear to decrease, but the difference was not statistically significant, during the occlusion period of the hyperaemia reactive test in the PROP-M group after treatment. This response has been associated with an improved microcirculatory response due to enhanced oxygen delivery and mitochondrial utilisation [46]. Previous studies using PROP in the form of a dietary supplement showed a decrease in BP in rats [26,27] and hypertensive individuals [28]. However, the current study is the first to indicate a BP-lowering effect of PROP when administered in the form of a mouthwash. This intriguing physiological response of PROP could not be attributed to oral microbiome changes. Previous studies suggested that the consumption of PROP increased NO availability due to the upregulation of eNOS expression [29]. Our results, showing a significant increase of plasma nitrate, align with this perspective. However, it is currently difficult to explain these physiological changes from a mechanistic perspective and how a local oral treatment could induce an increase in circulatory nitrate levels without increasing this anion in saliva.

This study had some limitations worth highlighting. It is likely that higher concentrations of propolis (>2.5%) are needed to promote an antimicrobial effect. Furthermore, the composition of PROP can vary based on the geographical location, plant species, environmental conditions and the species of bee [25]. This poses a challenge for pharmaceutical and oral care companies when attempting to develop new products based on natural compounds like PROP, as they need to meet regulations from official bodies such as the U.S. Food and Drug Administration (FDA) or the European Medicines Agency (EMA). Additionally, this study had a small sample size that was predominantly female. We recognise that future studies should better match the number of males and females to provide stronger insights about the intervention in both sexes. Another limitation of this study was the lack of a placebo group which could have helped control for potential confounding variables and allowed to better isolate the specific effects of each treatment on the microbiome. Furthermore, the small reduction in systolic BP observed after using the PROP-M may have a lower clinical impact in terms of cardiovascular risk, however, it remains to be elucidated whether this hypotensive response may be greater in individuals with high BP. Finally, this study was conducted during the COVID-19 pandemic, which was also an important limitation for undertaking oral examinations in all the participants from this study.

## Conclusions

This study showed for the first time that PROP-M at low concentrations (2.5%) had a limited effect in modifying the relative abundance of NPS and their NPA. However, PROP-M exhibited positive effects by increasing plasma nitrate availability and reducing systolic BP.

Conversely, CHX-M caused substantial changes in the oral microbiome, including an increase in the relative abundance of NPS. However, consistent with our previous studies, we found a significant decrease in NPA that was accompanied by alterations in saliva composition towards an acidified oral environment. These results highlight the vital role of the activity of NPS in maintaining oral health.

Overall, these findings may have potential clinical implications. While PROP-M showed potential as an adjunct oral therapy for hypertensive patients, caution may be needed when pre-escribing CHX-M, particularly at high doses (0.2%), to similar patients, as it can induce oral microbial dysbiosis leading to lower nitrite bioavailability.

## Supplementary Material

Supplementary_dataclean.docx
