# Supplementary material for: The comparative effect of propolis and chlorhexidine mouthwash on oral nitrite-producing bacteria and blood pressure regulation
Source: J Oral Microbiol. 2024 Dec 16;17(1):2439636. doi: 10.1080/20002297.2024.2439636 (PMC11650436; doi:10.1080/20002297.2024.2439636)
Supplement: Supplementary_dataclean.docx [file ZJOM_A_2439636_SM4583.docx]

**Appendix A: Measurement of the microvascular function using a flow-mediated test**

Levels of oxygenated haemoglobin (HbO_2_) and deoxyhaemoglobin (HHb) on the left forearm (extensor digitorum) were continuously recorded using a near infra-red spectroscopy (NIRS) device (NIRO-200NX, Hamamatsu, Japan) at an output frequency of 1 Hz. After baseline measurements (2 min), an automatic pneumatic cuff (Hokanson E-20 AG101, USA) was inflated ~5 cm above the elbow for 5 min to an occlusion pressure of 200 mmHg. Then, inflation of the cuff was rapidly released (< 1 second) and the NIRS measurements were continuously monitored for 5 more minutes. Several NIRS measurements were analysed:

(I) Baseline value, the average value during the first 2 min of the test.

(II) Area under the curve 1 (AUC 1), the area above the slope during occlusion and baseline levels.

(III) Occlusion: ∆min/baseline, the difference between baseline and the minimal value reached during the ischemia phase as an index of muscle oxygen consumption.

(IV) Reperfusion: ∆max/baseline, the difference between baseline and the maximal value reached during the reperfusion phase.

(V) Area under the curve 2 (AUC 2), the area below the recovery and baseline levels.

**
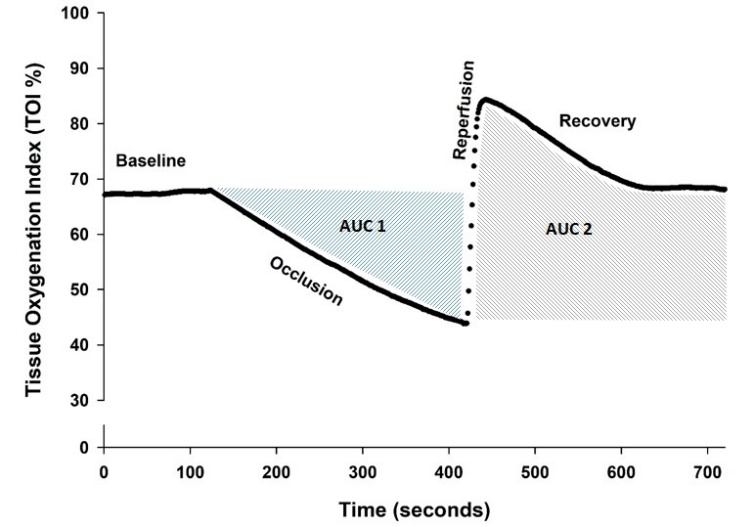
**

Figure Appendix 1. Microvascular response to a hyperaemia reactive test including the baseline period, the occlusion period (AUC 1, and ∆min/baseline) the reperfusion period (∆max/baseline), and the recovery period (AUC 2).

**Appendix B: DNA extraction, sequencing and analyses**

Saliva pellets were used for the analysis of the microbiome. The ZymoBIOMICS® Gut Microbiome Standard (ref #D6331) was used as a positive control. DNA extraction was performed with the ZymoBIOMICS 96 MagBead DNA kit (ref #D4302, ZymoResearch), using the FastPrep-96 and 32-PurePrep (MolGen).

A deep-well plate was prepared containing the following solutions of the kit: columns 1 and 7: 600 μl MagBinding buffer, 25 μl of MagBinding Beads; columns 2 and 8: 500 μl MagBinding buffer; columns 3 and 9: 500 μl MagWash 1; columns 4, 5, 10 and 11: 900 μl MagWash 2, and column 6 and 12: 50 μl Elution buffer.

Saliva pellets were resuspended in 800μl Lysis solution of the kit and 800μl were transfered into a ZymoResearch BashingBead™ Lysis Tube. These tubes were agitated in FastPrep-96 at max speed (1,800 rpm) for 5 minutes and then centrifuged at 5,000 g for 5 minutes. 200μl of the supernatant were added to columns 1 and 7 of the above mentioned prepared deep-well plate.

The plate was introduced in the 32-PurePrep machine, with the following configuration:

1. STEP 1/WELL 1: LyseBind. Mix time: 10 minutes. Magnet: 60 seconds. Wait Time: 0 minutes. Volume: 825 μl. Mix Speed (1-10): 10. Temperature: OFF.
2. STEP 2/WELL 2: MagBind. Mix time: 1 minute. Magnet: 30 seconds. Wait Time: 0 minutes. Volume: 500 μl. Mix Speed (1-10): 8. Temperature: OFF.
3. STEP 3/WELL 3: MagWash1. Mix time: 1 minute. Magnet: 30 seconds. Wait Time: 0 minutes. Volume: 500 μl. Mix Speed (1-10): 8. Temperature: OFF.
4. STEP 4/WELL 4: MagWash2. Mix time: 1 minute. Magnet: 30 seconds. Wait Time: 20 minutes. Volume: 900 μl. Mix Speed (1-10): 8. Temperature: OFF.
5. STEP 5/WELL 5: MagWash2. Mix time: 1 minute. Magnet: 30 seconds. Wait Time: 0 minutes. Volume: 900 μl. Mix Speed (1-10): 8. Temperature: OFF.
6. STEP 6/WELL 6: Elution. Mix time: 10 minutes. Magnet: 60 seconds. Wait Time: 0 minutes. Volume: 50 μl. Mix Speed (1-10): 5. Temperature: OFF.
7. STEP 7/WELL 5: Discard. Mix time: 1 minute. Magnet: 0 seconds. Wait Time: 0 minutes. Volume: 900 μl. Mix Speed (1-10): 8. Temperature: OFF.

After this step, the eluted samples at columns 6 and 12 were transfer to a 96 well plate to proceed to 16S amplicon sequencing. The V3-V4 region of the bacterial 16S ribosomal RNA gene was amplified using the following universal primers in a limited cycle PCR: V3-V4-Forward (5′-TCGTCGGCAGCGTCAGATGTGTATAAGAGACAGCCTACGGGNGGCWGCAG-3′) and V3-V4-Reverse (5′-GTCTCGTGGGCTCGGAGATGTGTATAAGAGACAGGACTACHVGGGTATCTAATCC-3′). Then full-length Nextera adapters with barcodes for multiplex sequencing were added in a second PCR step, resulting in sequencing ready libraries. Sequencing was performed in the Illumina MiSeq with 2 × 300 bp reads using v3 chemistry at the Genomics facility of the Centre for Genomic Regulation (CRG, Barcelona). Two bacterial mock communities from the BEI Resources of the Human Microbiome Project (HM-276D and HM-277D) were amplified and sequenced in the same manner as all other samples. Negative controls of PCR amplification were also included in parallel, using the same conditions and reagents.

Microbial community analyses were performed with R v4.3.1. Dada2 v1.12.1 was used for quality filtering, with parameters ‘truncLen = c(270, 225), trimLeft = 10 minLen = 50, maxEE = 8, maxN = 0’. Dada2 was also used for ASV clustering, chimera removal and taxonomic assignment of ASVs with the database SILVA nr99 v138.1. Phyloseq v1.44 was used to analyse the abundance tables. Abundance tables were 0-replaced using the CZM method in zCompositions v1.4.0.1 and then CLR-transformed with CoDaSeq v0.99.6. Differential abundance analyses were performed using mixed-effect linear models with lmer4 v1.1.33. When comparing paired samples, the subject was added as a random effect. P-values for the multiple tests were adjusted using the Benjamini-Hochberg correction (FDR correction.)

List of potential and confirmed nitrite-producing species of the oral microbiota. (Taken from Rosier B et al. The importance of nitrate reduction for oral health. J Dent Res 2022; 101(8): 887-897.

| *Actinomyces georgiae* |
| --- |
| *Actinomyces graevenitzii* |
| *Actinomyces hongkongensis* |
| *Actinomyces johnsonii* |
| *Actinomyces lingnae* |
| *Actinomyces massiliensis* |
| *Actinomyces naeslundii* |
| *Actinomyces odontolyticus* |
| *Actinomyces oris* |
| *Actinomyces viscosus* |
| *Capnocytophaga gingivalis* |
| *Capnocytophaga ochracea* |
| *Capnocytophaga sputigena* |
| *Corynebacterium durum* |
| *Corynebacterium matruchotii* |
| *Cutibacterium acnes* |
| *Eikenella corrodens* |
| *Fusobacterium nucleatum* |
| *Granulicatella adiacens* |
| *Haemophilus parainfluenzae* |
| *Haemophilus segnis* |
| *Kingella denitrificans* |
| *Neisseria elongate* |
| *Neisseria flavescens* |
| *Neisseria macacae* |
| *Neisseria mucosa* |
| *Neisseria oralis* |
| *Neisseria sicca* |
| *Neisseria subflava* |
| *Paraburkholderia fungorum* |
| *Prevotella melaninogenica* |
| *Propionibacterium acnes* |
| *Pseudopropionibacterium propionicum* |
| *Rothia aeria* |
| *Rothia dentocariosa* |
| *Rothia mucilaginosa* |
| *Schaalia odontolytica* |
| *Selenomonas artemidis* |
| *Selenomonas flueggei* |
| *Selenomonas noxia* |
| *Streptococcus australis* |
| *Streptococcus infantis* |
| *Streptococcus mitis* |
| *Streptococcus mutans* |
| *Streptococcus oralis* |
| *Streptococcus parasanguinis* |
| *Streptococcus salivarius* |
| *Streptococcus sanguinis* |
| *Veillonella atypica* |
| *Veillonella dispar* |
| *Veillonella parvula* |
| *Veillonella tobetsuensis* |

List of species associated with periodontitis. (Take from [Chen T et al. SMDI: An Index for Measuring Subgingival Microbial Dysbiosis. J Dent Res. 2022 Mar;101(3):331-338)](https://pubmed.ncbi.nlm.nih.gov/34428955/)

| *Actinomyces naeslundii* |
| --- |
| *Alloprevotella rava* |
| *Alloprevotella tannerae* |
| *Anaeroglobus geminatus* |
| *Atopobium rimae* |
| *Campylobacter concisus* |
| *Campylobacter gracilis* |
| *Campylobacter rectus* |
| *Campylobacter showae* |
| *Capnocytophaga gingivalis* |
| *Capnocytophaga granulosa* |
| *Capnocytophaga leadbetteri* |
| *Capnocytophaga sputigena* |
| *Catonella morbi* |
| *Corynebacterium matruchotii* |
| *Dialister invisus* |
| *Dialister pneumosintes* |
| *Eikenella corrodens* |
| *Filifactor alocis* |
| *Fretibacterium fastidiosum* |
| *Fusobacterium naviforme* |
| *Fusobacterium naviformenucleatum* |
| *Fusobacterium nucleatum* |
| *Fusobacterium nucleatum* |
| *Fusobacterium nucleatum* |
| *Fusobacterium nucleatum* |
| *Fusobacterium periodonticum* |
| *Fusobacterium sp. HOT 204* |
| *Gemella morbillorum* |
| *Granulicatella adiacens* |
| *Haemophilus parainfluenzae* |
| *Kingella oralis* |
| *Lachnoanaerobaculum saburreum* |
| *Lautropia mirabilis* |
| *Leptotrichia buccalis* |
| *Leptotrichia hofstadii* |
| *Leptotrichia hongkongensis* |
| *Leptotrichia wadei* |
| *Mogibacterium diversum* |
| *Mogibacterium timidum* |
| *Mycoplasma faucium* |
| *Mycoplasma salivarium* |
| *Neisseria elongata* |
| *Neisseria subflava* |
| *Parvimonas micra* |
| *Peptostreptococcus stomatis* |
| *Porphyromonas endodontalis* |
| *Porphyromonas gingivalis* |
| *Prevotella dentalis* |
| *Prevotella denticola* |
| *Prevotella intermedia* |
| *Prevotella maculosa* |
| *Prevotella melaninogenica* |
| *Prevotella nigrescens* |
| *Prevotella oralis* |
| *Prevotella oris* |
| *Prevotella oulorum* |
| *Prevotella pleuritidis* |
| *Rothia aeria* |
| *Rothia dentocariosa* |
| *Selenomonas noxia* |
| *Selenomonas sputigena* |
| *Shewanella algaehaliotisupenei* |
| *Solobacterium moorei* |
| *Stomatobaculum longum* |
| *Streptococcus anginosus* |
| *Streptococcus constellatus* |
| *Streptococcus cristatus* |
| *Streptococcus dentisani* |
| *Streptococcus gordonii* |
| *Streptococcus intermedius* |
| *Streptococcus mitis* |
| *Streptococcus parasanguinis* |
| *Streptococcus sanguinis* |
| *Streptococcus tigurinus* |
| *Tannerella forsythia* |
| *Treponema denticola* |
| *Treponema lecithinolyticum* |
| *Treponema maltophilum* |
| *Treponema medium* |
| *Treponema socranskii* |
| *Veillonella parvula group* |

**Appendix C: Antibacterial activity analyses and results**

*Media*

Brain Heart Infusion (BHI) Broth (Merck, Dorset, UK) was made by adding 37g/L, based on the manufacturer’s instructions. Double strength BHI broth (DS BHI) was made by adding 74g/L. BHI agar plates were made with BHI Broth supplemented with 1.5% v/w agarose No.1 (Neogen, Auchincruive, Scotland). All media was autoclaved at 121°C, at 15 psi, for 15 minutes.

*Bacterial culture*

*Rothia dentocariosa* DSM 43762 and *Streptococcus mutans* NCIMB 702062 were maintained on Brain Heart Infusion (BHI) agar plates, incubated at 37°C in air + 5% CO^­^_2_ for 24 hours. For liquid cultures, one colony was transferred into 10ml BHI broth in a 30 ml universal tube and incubated at 37°C in air + 5% CO^­^_2_ for 24 hours, shaking at 180 rpm in a rack angled at 45° (from the vertical), with the universal lid loose and held in place with masking tape to allow gas transfer.

*Propolis (PROP) assay*

To test the effect of PROP on the growth of *R. dentocariosa* and *S. mutans*, six universal tubes were set up as follows: 1) *R. dentocariosa* + 20% propolis, 2) *S. mutans* + 20% PROP, 3) *R. dentocariosa* positive control, 4) *S. mutans* positive control, 5) BHI negative control, 6) BHI + 20% propolis negative control (Table S1). Tubes were vortexed and incubated as previously described. Every two hours (0, 2, 4, 6, and 8 hours) plus 24 hours, the tubes were taken from the incubator, vortexed, and a 100µl aliquot removed and serially diluted into 900µl phosphate buffered saline down to 10^-9^, using a 2ml, 96-well round bottom deep well plate (Axygen, Fisher Scientific, Loughborough, UK). Using a modified version of the Miles and Misra method (Miles et al, 1938), viable counts were performed by transferring 3 x 5µl of each dilution to BHI agar using a multichannel pipette and incubated at 37°C in air + 5% CO^­^_2_ for 24 hours. Colony forming units were counted at the appropriate dilution and an average of the three 5µl volumes calculated. Four biological repeats (n=4) were performed.

Table Appendix 1. Set up of the propolis (PROP) assay for *R. dentocariosa* and *S. mutans* plus the controls.

| Tube | DS BHI (µl) | PROP  (µl) | Sterile water (µl) | BHI (µl) | Overnight Bacterial culture (µl) |
| --- | --- | --- | --- | --- | --- |
| *R. dentocariosa* + 20% propolis | 750 | 300 | 375 |  | 75 |
| *S. mutans* + 20% propolis | 750 | 300 | 375 |  | 75 |
| *R. dentocariosa* positive control | 750 | - | 675 |  | 75 |
| *S. mutans* positive control | 750 | - | 675 |  | 75 |
| BHI negative control | 750 | - | 675 | 75 |  |
| BHI + 20% propolis negative control | 750 | 300 | 375 | 75 |  |

**Results**

Culturing of *R. dentocariosa* in BHI broth produced a standard growth curve, with an average final colony count at 24-hours of 1.28 x 10^10^ CFU/ml (Figure S1), with a range of between 3.07 x 10^9^ and 2.8 x 10^10^ CFU/ml. No growth was observed in the BHI negative control. When cultured in BHI broth plus 20% PROP, the growth of *R. dentocariosa* was found to be repressed, with an initial average inoculation at time zero of 1.07 x 10^8^ CFU/ml (between 9.33 x 10^6^ and 2.8 x 10^8^ CFU/ml), with counts reducing to an average of 3.17 x 10^4^ CFU/ml at the final 24-hour time point (between 0 and 4.67 x 10^6^ CFU/ml).


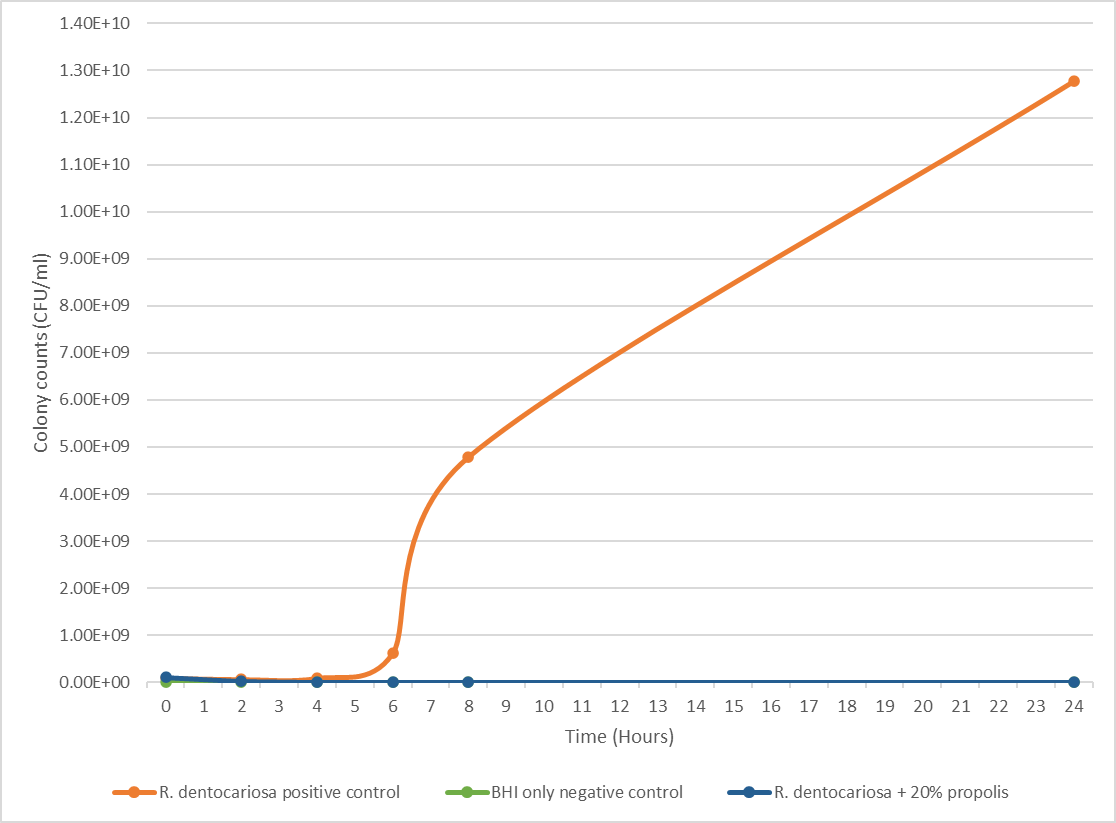


Figure Appendix 2. The effect of 20% PROP on the growth of *R. dentocariosa* in BHI broth over a 24-hour period, measured by colony counts averaged from four biological replicated (n=4).

A similar trend was observed for *S. mutans*, with an average final colony count at 24-hours of 5.89 x 10^8^ CFU/ml (Figure S2), with a range of between 2.53 x 10^7^ and 1.47 x 10^9^ CFU/ml for the positive control. No growth was observed in the BHI negative control. When cultured in BHI broth plus 20% propolis, the growth of *S. mutans* was similarly repressed, with an average of 5.37 x 10^5^ CFU/ml and a range of between 0 and 1.67 x 10^6^ CFU/ml.


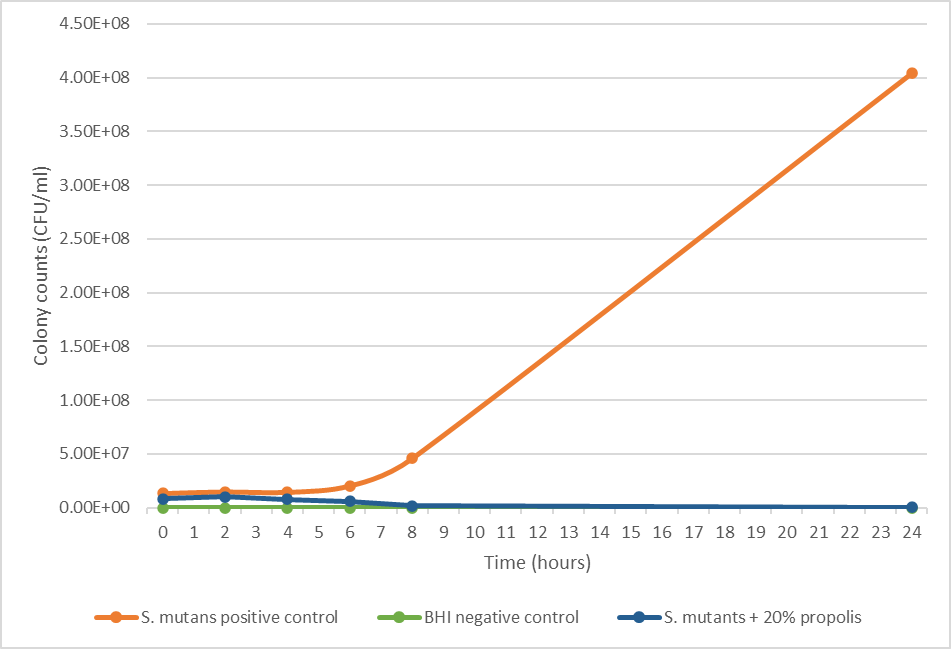


Figure Appendix 3. The effect of 20% PROP on the growth of *S. mutans* in BHI broth over a 24-hour period, measured by colony counts averaged from three biological replicates (n=3).

**Appendix D: Detection of phenolic compounds in the PROP-M**


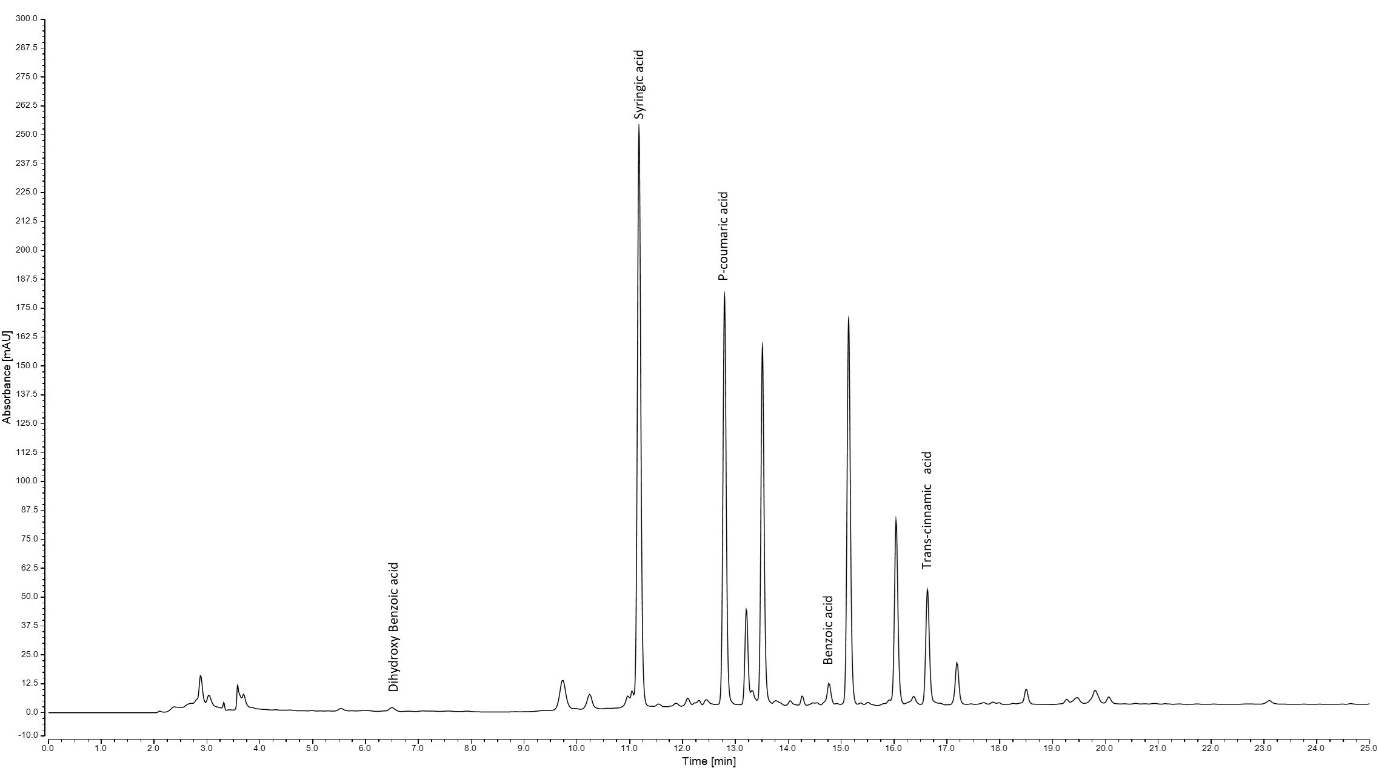


Figure Appendix 5: Five phenolic compounds were detected; dihydroxy benzoic acid, syringic acid, p-coumaric acid main, benzoic acid, trans-cinnamic acid.

**Appendix E: Results of dental plaque and bleeding scores**


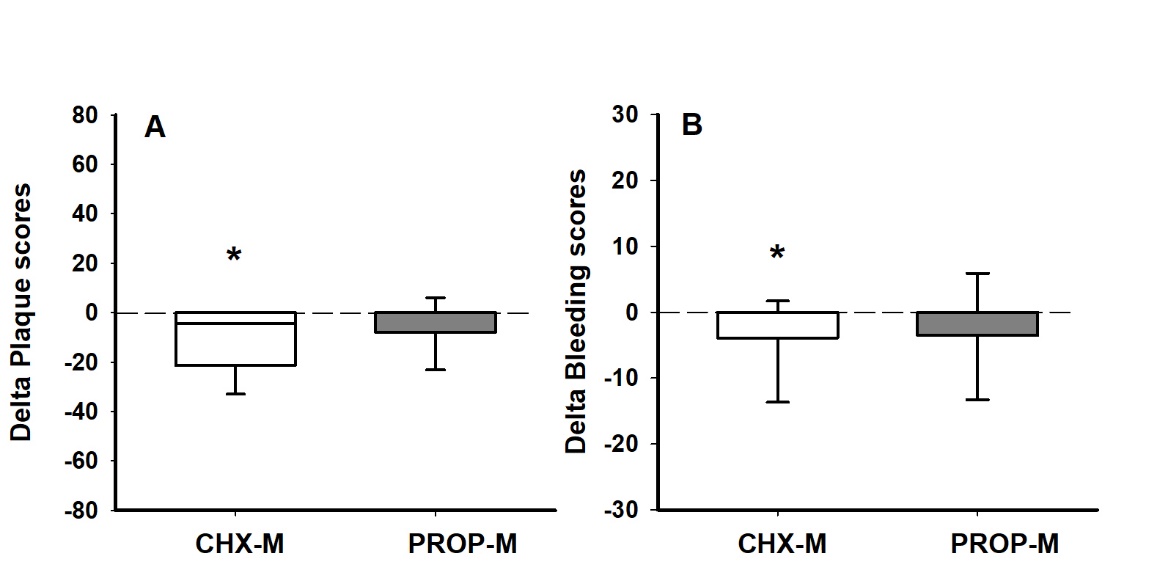


Figure Appendix 4. Difference (mean ± SD) in O’Leary dental plaque (5A) and bleeding (5B) scores after chlorhexidine (CHX-M; n=13) and propolis (PRO-M; n=12) treatment. (* represents statistical differences between pre and post measurements within the same group).


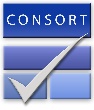
CONSORT 2010 checklist of information to include when reporting a randomised trial*

| Section/Topic | Item No | Checklist item | Reported on page No |
| --- | --- | --- | --- |
| Title and abstract | | | |
|  | 1a | Identification as a randomised trial in the title | 2 |
|  | 1b | Structured summary of trial design, methods, results, and conclusions (for specific guidance see CONSORT for abstracts) | 2 |
| Introduction | | | |
| Background and objectives | 2a | Scientific background and explanation of rationale | 2-3 |
|  | 2b | Specific objectives or hypotheses | 3 |
| Methods | | | |
| Trial design | 3a | Description of trial design (such as parallel, factorial) including allocation ratio | 4 |
|  | 3b | Important changes to methods after trial commencement (such as eligibility criteria), with reasons | 4 |
| Participants | 4a | Eligibility criteria for participants | 4 |
|  | 4b | Settings and locations where the data were collected | 4 |
| Interventions | 5 | The interventions for each group with sufficient details to allow replication, including how and when they were actually administered | 4-5 |
| Outcomes | 6a | Completely defined pre-specified primary and secondary outcome measures, including how and when they were assessed | 5-6 |
|  | 6b | Any changes to trial outcomes after the trial commenced, with reasons |  |
| Sample size | 7a | How sample size was determined | 4 |
|  | 7b | When applicable, explanation of any interim analyses and stopping guidelines |  |
| Randomisation: |  |  |  |
| Sequence generation | 8a | Method used to generate the random allocation sequence | 5 |
|  | 8b | Type of randomisation; details of any restriction (such as blocking and block size) | 5 |
| Allocation concealment mechanism | 9 | Mechanism used to implement the random allocation sequence (such as sequentially numbered containers), describing any steps taken to conceal the sequence until interventions were assigned | 5 |
| Implementation | 10 | Who generated the random allocation sequence, who enrolled participants, and who assigned participants to interventions | 5 |
| Blinding | 11a | If done, who was blinded after assignment to interventions (for example, participants, care providers, those assessing outcomes) and how | 4 |
|  | 11b | If relevant, description of the similarity of interventions |  |
| Statistical methods | 12a | Statistical methods used to compare groups for primary and secondary outcomes | 5-6 |
|  | 12b | Methods for additional analyses, such as subgroup analyses and adjusted analyses |  |
| Results | | | |
| Participant flow (a diagram is strongly recommended) | 13a | For each group, the numbers of participants who were randomly assigned, received intended treatment, and were analysed for the primary outcome | 6 |
|  | 13b | For each group, losses and exclusions after randomisation, together with reasons | 7 |
| Recruitment | 14a | Dates defining the periods of recruitment and follow-up | 6 |
|  | 14b | Why the trial ended or was stopped |  |
| Baseline data | 15 | A table showing baseline demographic and clinical characteristics for each group | Table 1 |
| Numbers analysed | 16 | For each group, number of participants (denominator) included in each analysis and whether the analysis was by original assigned groups | 6 |
| Outcomes and estimation | 17a | For each primary and secondary outcome, results for each group, and the estimated effect size and its precision (such as 95% confidence interval) |  |
|  | 17b | For binary outcomes, presentation of both absolute and relative effect sizes is recommended |  |
| Ancillary analyses | 18 | Results of any other analyses performed, including subgroup analyses and adjusted analyses, distinguishing pre-specified from exploratory |  |
| Harms | 19 | All important harms or unintended effects in each group (for specific guidance see CONSORT for harms) | 6 |
| Discussion | | | |
| Limitations | 20 | Trial limitations, addressing sources of potential bias, imprecision, and, if relevant, multiplicity of analyses | 11 |
| Generalisability | 21 | Generalisability (external validity, applicability) of the trial findings | 11 |
| Interpretation | 22 | Interpretation consistent with results, balancing benefits and harms, and considering other relevant evidence | 9-11 |
| Other information | | |  |
| Registration | 23 | Registration number and name of trial registry | 4 |
| Protocol | 24 | Where the full trial protocol can be accessed, if available |  |
| Funding | 25 | Sources of funding and other support (such as supply of drugs), role of funders | 12 |

Citation: Schulz KF, Altman DG, Moher D, for the CONSORT Group. CONSORT 2010 Statement: updated guidelines for reporting parallel group randomised trials. BMC Medicine. 2010;8:18.
© 2010 Schulz et al. This is an Open Access article distributed under the terms of the Creative Commons Attribution License (<http://creativecommons.org/licenses/by/2.0>), which permits unrestricted use, distribution, and reproduction in any medium, provided the original work is properly cited.

*We strongly recommend reading this statement in conjunction with the CONSORT 2010 Explanation and Elaboration for important clarifications on all the items. If relevant, we also recommend reading CONSORT extensions for cluster randomised trials, non-inferiority and equivalence trials, non-pharmacological treatments, herbal interventions, and pragmatic trials. Additional extensions are forthcoming: for those and for up-to-date references relevant to this checklist, see [www.consort-statement.org](http://www.consort-statement.org).
